# Patient involvement for improved patient safety: A qualitative study of nurses’ perceptions and experiences

**DOI:** 10.1002/nop2.89

**Published:** 2017-08-29

**Authors:** Janna Skagerström, Carin Ericsson, Per Nilsen, Mirjam Ekstedt, Kristina Schildmeijer

**Affiliations:** ^1^ Research and Development Unit in Region Östergötland and Department of Medical and Health Sciences Linköping University Linköping Sweden; ^2^ Centre of Heart and Medicine Region Östergötland Linköping Sweden; ^3^ Department of Medical and Health Sciences Linköping University Linköping Sweden; ^4^ Department of Health and Caring Sciences Linnaeus University Kalmar Sweden; ^5^ Department of Learning, Informatics, Management and Ethics Karolinska Institutet Stockholm Sweden

**Keywords:** barriers, determinants, facilitators, nurses, patient involvement, patient safety

## Abstract

**Aim:**

To explore nurses’ perceptions and experiences of patient involvement relevant to patient safety.

**Design:**

Qualitative design using individual semi‐structured interviews.

**Methods:**

Interviews with registered nurses (*n *=* *11) and nurse assistants (*n *=* *8) were conducted in 2015–2016. Nurses were recruited from five different healthcare units in Sweden. The material was analysed using conventional content analysis.

**Results:**

The analysis resulted in four categories: healthcare professionals’ ways of influencing patient involvement for safer care; patients’ ways of influencing patient involvement for safer care; barriers to patient involvement for safer care; and relevance of patient involvement for safer care. The nurses expressed that patient involvement is a shared responsibility. They also emphasized that healthcare provider has a responsibility to create opportunities for the patient to participate. According to the nurses, involvement can be hindered by factors related to the patient, the healthcare provider and the healthcare system. However, respondents expressed that patient involvement can lead to safer care and benefits for individual patients.

**Why is this research needed?**

- The patient has an overall perspective of their care and observes the whole care process.
- Most research on patient involvement concern the patient perspective and there is limited knowledge about the healthcare professionals’ views on patient participation for patient safety.

**What are the key findings?**

- The nurses believed that healthcare professionals and patients had a shared responsibility for patient participation to occur.
- The nurses emphasized the importance of their own initiatives to achieve patient involvement for enhanced patient safety by initiating dialogue and inviting the patients to ask questions.
- The nurses expressed that barriers to achieve patient participation for safer care were seen both within patients, healthcare professionals and the healthcare system.

**How should the findings be used to influence policy/practice/research/education?**

- The healthcare system should allocate time and supportive environments to facilitate open dialogue between healthcare professionals and patients.
- Healthcare professionals should be offered training in how to encourage the patients to be involved in their health care.

## 1 INTRODUCTION

Patient safety has progressed over the last 15 years from being a relatively insignificant issue to a position high on the agenda for healthcare professionals, managers and policy makers as well as the public. Sweden has seen increased patient safety efforts since 2009 when a national study on adverse events in Swedish hospital care was published (Soop, Fyksmark, Köster, & Haglund, 2009). The study estimated the percentage of preventable adverse events as high as 8.6% in hospital care, demonstrating that the magnitude of the patient safety problem was not smaller in Sweden than elsewhere. Efforts for improved patient safety in Sweden were further enhanced in 2011 with the introduction of a new law on patient safety and a financial incentive for county councils (responsible for providing health care in Sweden) that performed certain patient safety‐enhancing activities (Ridelberg, Roback, Nilsen, & Carlfjord, 2016).

There is increasing interest in involving patients in safety‐related initiatives, premised on the assumption that their interaction with healthcare professionals can improve the safety of health care in many ways (Berger, Flickinger, Pfoh, Martinez, & Dy, 2014, World Health Organization, 2013aa). The importance of eliciting and acting on patients’ concerns has been emphasized. The patients are privileged witnesses of health care because they are at the centre of the process of care and observe the whole process (Schwappach & Wernli, 2010). Patients also carry out hidden work to compensate for inefficiencies of the healthcare system, such as relaying information between healthcare professionals (Vincent & Davis, 2012). Various policy initiatives have been undertaken aimed at encouraging patients in a range of safety‐relevant behaviours. The World Health Organization (WHO) promotes the program “Patients for Patient Safety” to bring together patients and various stakeholders to improve patient safety through advocacy, collaboration and partnership (WHO, 2013). In Sweden, the National Board of Health and Welfare and Swedish Association of Local Authorities and Region (SALAR), representing the county councils and municipalities, have emphasized the importance of a new perspective on the patient for improved quality and effectiveness of health care (National Board of Health and Welfare, 2015, SALAR, 2011). Healthcare professionals are also obliged by the law to give patients an opportunity to take part in patient safety work (SFS, 2010).

### 1.1 Background

Research indicates that there is a potential for patients to improve safety (Davis, Jacklin, Sevdalis, & Vincent, 2007; Vincent & Coulter, 2002) and that patients are willing and able to be involved in safety‐related work (Waterman et al., 2006 Wright et al., 2016). However, several barriers to involving patients in improving patient safety has been identified and organized into three key barriers: (i) patients are not always willing or prepared to commit their time and energy to improve their care because they have enough to worry about being ill; (ii) healthcare professionals represent traditional medical authority and questioning or advising professionals about what they do is unacceptable for many patients; and (iii) patients may be apprehensive about reporting problems in their care when providers’ responses are unappreciative or when the patients believe that their feedback may jeopardize the providers’ goodwill towards the patient (Iedema, Allen, Britton, & Gallagher, 2012). Organizational factors such as a busy setting, lack of continuity of care and patients being unaware of incident reporting systems have also been identified as barriers to active patient participation (Doherty & Stavropoulou, 2012).

Nurses comprise the largest professional group in health care in Sweden. The main categories of nurses in Swedish health care are registered nurses and nurse assistants, who differ with regard to their level of education, work duties and responsibilities. Registered nurses are critically important to achieve patient safety since they often have a role as coordinator of multidisciplinary care and are involved with many aspects of patient care, from providing comfort and hygiene to administering injections, updating medical records, as well as handling some therapeutic and diagnostic procedures. Several studies have stressed the importance of nurses’ role for identifying, interrupting and correcting medical adverse events (Gaffney, Hatcher, & Milligan, 2016) and for reducing patients’ feelings of being unsafe and vulnerable in the health care setting (Kenward, Whiffin, & Spalek, 2017).

Thus far, very few studies have investigated nurses and other healthcare professionals’ attitudes, beliefs and behaviours concerning patient involvement for improved patient safety. Research conducted hitherto suggests that providers may be willing to support patient involvement in safety‐relevant behaviours, although the factors behind these preliminary findings remain largely unexplored (Davis, Briggs, Arora, Moss, & Schwappach, 2014; Hochreutener & Wernli, 2010; Schwappach, Frank, & Davis, 2012). A previous Swedish study assessing nurses’ perceptions of factors influencing patient safety found that patient‐nurse interaction was an important factor that could hinder or facilitate enhanced patient safety depending on the quality of the communication (Ridelberg, Roback, & Nilsen, 2014). This study provides an in‐depth investigation into nurses’ perspectives on patient involvement for safer care in meetings with healthcare professionals, being the first Nordic study on this topic. The aim was to explore nurses’ experiences and perceptions with regard to patient involvement of relevance for patient safety.

## 2 THE STUDY

### 2.1 Study setting

The study was set in Sweden. Health care in Sweden is mainly tax‐funded although private health care also exists. All residents are insured by the state, with equal access for the entire population. Out‐of‐pocket fees are low and regulated by law. The responsibility for health and medical care in Sweden is shared by the central government, county councils and municipalities throughout Sweden. The health care system is financed primarily through taxes levied by county councils and municipalities.

### 2.2 Study design

A qualitative study approach using standardized (also referred to as structured) open‐ended interviews was deemed appropriate regarding the explorative aim of the study. This qualitative descriptive study is grounded in the assumption that human beings construct the meaning of their experiences in social interaction with their environment. Qualitative descriptive studies comprise a valuable methodologic approach, by using open‐ended interviews where the phenomenon under study is explored in an interaction between the interviewer and the interviewee (Sandelowski, 2000).

### 2.3 Participants

We used a purposeful sampling strategy to achieve a heterogeneous sample of nurses working in different healthcare facilities, with patients who varied in terms of health status (from patients seen in primary health care to ill patients receiving hospital care and, for example, surgery patients), length of stay in health care (from patients visiting outpatient facilities to in‐hospital patients) and age. The aim was to achieve a sample of nurses that represented a broad spectrum of perceptions and experiences concerning patient involvement in relation to patient safety.

The nurses were recruited using an email that briefly described the study. The email request was sent to the manager of each work unit, explaining that we wanted a sample of three or four nurses. The manager in turn forwarded the request to all or a sample of registered nurses and nurse assistants at the unit. An information letter describing the study was sent to interested nurses and the interviews were scheduled. No respondents declined involvement after receiving the information letter.

### 2.4 Data collection

The interview guide used in the study was developed by the authors and concerned the nurses’ experiences and perceptions regarding patient involvement of relevance for patient safety. There were general questions on patient involvement of relevance for patient safety. There were also specific questions on the respondent's own experiences and examples of patients who have observed and highlighted something of importance for patient safety. The interview guide ended with questions on existing routines to account for patients’ views and experiences and on the nurses’ suggestions on how patient involvement for safer care can be achieved.

Patient safety was defined in accordance with the definition used in Swedish law (SFS, 2010), that is: “protection against adverse events” where adverse events is defined as “suffering, bodily or mental harm or illness and deaths that could have been avoided if adequate measures had been taken at the patient contact with the healthcare system”. The definition was read to the nurses at the beginning of the interview and a printed definition was placed on the table during the interview so that the respondents could read it.

The questions were pilot tested in one test interview, not analysed. The test interview indicated that the questions were generic enough to be used in different healthcare contexts and that the wording was clear. The interviews were conducted by KS, CE and JS and were digitally recorded using a Dictaphone. Interviews were held during regular working hours to facilitate involvement. Each interview lasted between 18–53 min. The interviews were transcribed verbally by a firm specialized in transcription. The researchers checked the transcripts and removed statements that could reveal the identity of the informant.

Before starting the interviews, the participants were asked to re‐read the information letter and give their written informed consent to participate. Each interview started with an open question asking the participants to describe their thoughts on how patients can influence patient safety. The questions were open ended to stimulate narratives of the participants’ own experiences. During the interviews, probing questions were asked, for example: “what do you mean?” and “can you explain this a little further?” to deepen or clarify the descriptions or drawing the attention back to the topic (Kvale & Brinkman, 2009).

### 2.5 Data analysis

Data were analysed using content analysis. We followed the analytical procedure for conventional content analysis as detailed by Hsieh and Shannon (2005). The analysis was data driven and based on the participants’ unique perspectives rather than guided by a pre‐defined theory or hypothesis. Investigator triangulation was used to validate the findings. All researchers read and re‐read the transcripts to gain a sense of the content and an overview of the whole material. With the aim of the study in mind, the researchers highlighted text and made notes and headings in the margins to include all aspects of the content. Initial thoughts and impressions regarding the material were written down. No pre‐defined structures were used as the codes were derived from the data to capture key concepts. Codes that were related to each other were grouped and organized into subcategories and categories. This process was iterative, going back and forth checking the codes against the whole material. The subcategories and categories were subsequently compared for differences and similarities, with the aim of being as internally homogeneous and as externally heterogeneous as possible.

### 2.6 Rigour

Credibility in the data analysis was strengthened by the fact that the initial coding of the data was performed by several researchers independently (JS, CE and KS). The classification of categories and subcategories was then discussed by two researchers (JS and CE). After they reached consensus, the classification was discussed by all the authors and adjustments were made until all were satisfied. The multidisciplinary research team allowed different perspectives on the issue of patient involvement in relation to patient safety. The team consisted of a nurse with experience in clinical patient work as well as work with miscellaneous patient safety issues (KS), a public health researcher (JS), a behavioural science practitioner working with organizational development and experience in developing and implementing patient involvement policies (CE), a nurse experienced in qualitative methods, patient involvement and system safety issues (ME) and an experienced implementation and patient safety researcher (PN).

### 2.7 Ethical considerations

The study was performed according to the World Medical Association Declaration of Helsinki ethical principles for medical research involving human subjects. All the participants gave their consent to participate in the interviews. The study did not require ethical approval because it did not involve sensitive personal information, as specified in Swedish law regulating ethical approval for research concerning humans (SFS, 2003).

## 3 FINDINGS

Interviews were conducted with 19 nurses, of which 11 were registered nurses and 8 were nurse assistants. They were employed in five different work units: (i) pulmonary medical unit in a university hospital (550 beds); (ii) surgery unit in a mid‐sized hospital (350 beds), (iii) ear, nose and throat unit in a mid‐sized hospital (500 beds); (iv) one maternity care unit (outpatient care); and (v) one nursing home (18 residents). Table 1 provides information on the participants. The interviews were carried out from May 2015 – February 2016 at the participants’ work units.

**Table 1:** Participant characteristics

Characteristics	Registered nurses (*n *=* *11)	Nurse assistants (*n *=* *8)
Sex, *n*(%)		
Male	0 (0)	0 (0)
Female	11 (100)	8 (100)
Years of practice, *n*(%):		
0–1 years	0 (0)	0 (0)
2–4 years	1 (9)	2 (25)
5–9 years	1 (9)	0 (0)
10–20 years	7 (64)	2 (25)
21 years or more	2 (18)	4 (50)
Median years of practice, years	16	22
Years in the work unit, *n*(%)		
0–1 years	3 (27)	0 (0)
2–4 years	2 (18)	2 (25)
5–9 years	3 (27)	1 (13)
10–20 years	3 (27)	2 (25)
21 years or more	0 (0)	3 (38)
Median years in the work unit, years	5	10
Work unit, *n*(%):		
Pulmonary medicine unit	2 (18)	2 (25)
Surgical ward	2 (18)	2 (25)
Ear, nose and throat clinic	3 (27)	0 (0)
Maternity care centre	4 (36)	0 (0)
Nursing home	0 (0)	4 (50)

Analysis of the data yielded four categories related to patient involvement for enhanced patient safety: healthcare professionals’ ways of influencing patient involvement for safer care; patients’ ways of influencing patient involvement for safer care; barriers to patient involvement for safer care; and relevance of patient involvement for safer care (Table 2).

**Table 2:** Categories and subcategories

Category	Subcategory
Health care professionals’ initiatives to achieve patient involvement for safer care	Dialogue Information Trustful relationship
Patients’ initiatives to achieve patient involvement for safer care	Assuming responsibility for one's health and treatment Being active in communication with healthcare professionals
Interaction between healthcare professionals and patients to achieve patient involvement for safer care	Patients’ hesitancy to interact Constraints related to the healthcare system Healthcare professionals’ ambivalent feelings
Relevance of patient involvement for safer care	Patients receiving personal benefits Safer care

### 3.1 Healthcare professionals’ initiatives to achieve patient involvement for safer care

The nurses expressed that there were a few ways they and other healthcare professionals can influence patient involvement of potential relevance for patient safety. They believed that they could facilitate patient involvement by ensuring favourable conditions for dialogue with the patients, making sure that information is received and understood by the patients and creating a trustful relationship with the patients.

#### 3.1.1 Dialogue

The nurses described that they can facilitate patient involvement by providing conditions that are conducive to this involvement, including taking sufficient time to listen to patients and inviting them to ask questions and be active in the dialogue. Specific ways of achieving this included telling the patients that they are happy to answer any questions they might have, informing the patients that there will be time for their questions or concerns at the end of the consultation (after finishing medical examinations) and encouraging the patients to share their opinions regarding the health care:

Instead you have to be inviting and show a friendly response, encourage conversation and dialogue. You have to make sure it doesn't become a monologue, where you just sit and talk without… We, the staff, must encourage them to ask questions and to become involved. Participant 24

Some nurses mentioned that it is important to adapt to each individual patient they meet. It is especially important to be observant and take in facial expressions with patients who are unable to express themselves verbally.

#### 3.1.2 Information

The nurses expressed that they can influence the patients’ potential to be involved in their care by making sure that the patients receive and understand information provided to them. The information should be given in a language that can be understood by the patients and without medical terms that may be unfamiliar to the patients. The nurses mentioned that the patients’ abilities to assimilate information vary considerably and it may be necessary to repeat information at several time points. Patients with new diagnoses or treatments, patients with fatigue and patients discharged after a longer stay in hospital were all mentioned as groups that could benefit from repeated information:

Sometimes I think you could ask…”Do you think you got the information you needed, did you understand it?” or something like that, so it's not too much [information]. Participant 23

#### 3.1.3 Trustful relationship

The importance of a trustful relationship between the healthcare professional and patient to make the patients feel comfortable raising any concerns was made clear in the interviews. Although the nurses believed that the provider and patient have a shared concern for creating this relationship, the nurses argued that the ultimate responsibility to facilitate a trustful provider‐patient relationship rested with the providers of health care.

The importance of building a trustful relationship was primarily mentioned by nurses working in specialties which patients visit several times. Continuity of healthcare staff to ensure that the patient can meet the same professionals over time was mentioned as a factor that influenced the opportunity to establish a trusting relationship. The presence of a specific contact person to whom the patient could turn with their thoughts or questions was believed to enhance the patients’ confidence to engage in issues of potential relevance for patient safety.

Specific personal behaviours such as being empathic and humble as well as the ability to facilitate an open climate and allow sufficient time were seen as important to build a trustful relationship:

Yes, you have to be open, responsive in order for them [the patients] to open up. You can't just walk in and be really tough…that's not going to make it easy to open up if you have problems. Participant 16

### 3.2 Patients’ initiatives to achieve patient involvement for safer care

The nurses’ perceptions about what the patients can do differed somewhat depending on the healthcare context and what types of patients they typically meet. However, in general, nurses conveyed that the patients can assume responsibility for their health and treatment and be active in communication with healthcare professionals.

#### 3.2.1 Assuming responsibility for one's treatment and care

The nurses expressed that the patients can participate in their care and enhance patient safety by taking an active interest in their health and treatment. The interest could be manifested as searching for information or actively reading information. Further, using and asking for medical aids such as rollators, reading user manuals for medical devices used in home care or watching out for complications or abnormalities when in treatment were provided as examples of responsible patient actions to increase patient safety:

They [the patients] could get more involved in… to make sure things aren't forgotten, because we have a lot of different hoses and drainage, venous catheters and things like that, where they could help and be observant to prevent infections. Participant 4

#### 3.2.2 Being active in communication with healthcare professionals

Nurses stated that patients who are active in dialogue with healthcare professionals can improve patient safety. Writing down questions and thoughts or bringing a relative to appointments were tips for patients to prepare for communication with the professionals. Also, the nurses stated that the patients could be active by attending regular check‐ups, reminding staff about return visits or treatments and reporting any side effects:

They [the patients] have to tell us about, for example, side effects and things like that, that's nothing we can see ourselves. So, if I don't get that feedback, they might get medications that don't make them feel so good. Participant 3

Sharing detailed information about their medical conditions, heredity and side effects was viewed as important because this could help the healthcare professionals to understand the patients’ symptoms and healthcare needs and reduce the risk of important aspects being neglected:

Well, how it feels and… how they understand the situation, both physically and mentally, how they describe an ailment, how detailed they are… can actually make me reconsider and think otherwise. Participant 21

### 3.3 Interaction between healthcare professionals and patients to achieve patient involvement for safer care

The nurses were generally in favour of patient involvement and believed that it could lead to improved patient safety. However, they identified numerous potential problems and disadvantages associated with patient involvement, including problems relating to the patients’ lack of will and ability to participate, constraints related to the healthcare system and healthcare professionals’ ambivalent feelings concerning patient involvement.

#### 3.3.1 Patients’ hesitancy to interact

The nurses described that there are many obstacles to patients being active and participating in their care. They argued that some patients are unwilling to question healthcare professionals because they view them as authorities and reason that they, the doctor in particular, know what is best for them. Nurses believed that some patients might refrain from offering criticisms for fear of receiving suboptimal treatment or care. Patients who perceive that the healthcare professionals are stressed are unwilling to ask questions or start a dialogue because they feel that they might disturb or interrupt more important tasks:

When we seem stressed, they [the patients] feel they should not ask that simple question. You often hear that “I won't bother you [the staff] about this”. Participant 1

For some patients, participating in their treatment or care is hindered by health problems, difficulties with understanding, language problems or feeling uncomfortable with disclosing sensitive issues.

#### 3.3.2 Constraints related to the healthcare system

Several factors in the healthcare system were brought up by the nurses as hindering patient involvement to achieve safer care. Lack of privacy was a problem mentioned by nurses working in clinical wards where patients often share rooms. Shortage of the healthcare professionals’ time was another limitation for patient involvement. Appointments are sometimes just long enough for physical examinations but leave little time for dialogue or questions from the patients. The nurses thought that problems with availability and staff discontinuity can lead to disenchantment for the patients. Further, the possibility of building trustful relationships is decreased:

Temporary doctors mean that they [the patients] won't meet the same [doctor] next time and then they [the patients] say, “It's no use asking.” You often hear that. Participant 12

#### 3.3.3 Healthcare professionals’ ambivalent feelings

The nurses described a range of feelings towards active patients who are informed and may ask more critical questions. By and large, the nurses were pleased to learn from the patients. If they made a mistake, they were grateful that someone pointed it out to them, although the mistake itself could make them ashamed. Some informed and active patients could make the nurses feel incompetent or question their profession. Some nurses expressed concern that patients who question a great deal or want detailed information can take too much time:

They [the patients] have too little knowledge. At the same time, they want to be involved, which requires a lot… a sort of pedagogical responsibility rests with me that demands a lot [of time and energy]. Participant 21

### 3.4 Relevance of patient involvement for safer care

This category concerns the nurses’ perceptions of the “results” of patient involvement. Some of the nurses could not think of any example where a patient had recognized or reported something relevant for patient safety. They described situations where the patients’ involvement had not directly affected patient safety but had led to positive effects for the patients. Others shared examples of varying relevance for patient safety, for example, how patients’ involvement had directly prevented a mistake or eliminated potential patient safety hazards.

#### 3.4.1 Patients receiving personal benefits

The nurses believed that patients who were active and questioned aspects of their treatment or care, such as long waiting times or outdated medical aids, could gain advantages compared with patients who did not raise any complaints or concerns. Advantages such as getting help quicker, shorter waiting times for medical examinations or receiving a more modern type of medical aid were brought up in the interviews:

If you're active as a patient and ask when you can get an appointment that could definitely shorten the waiting time compared with if you remain quiet and wait. Participant 8

#### 3.4.2 Safer care

Several nurses shared examples of situations when involvement by patients led to improvements in patient safety. The examples included patients reminding about allergies, asking for aids to avoid fall injuries, observing defects in medical devices and asking about referrals that their healthcare provider had forgotten about:

There was one [patient] with coeliac disease who almost ate food that she should certainly not have. And, of course it was [detected] because she asked, “Is this really gluten‐free?” Participant 9

Another example of indirect patient involvement was when the nurses themselves thought of some hazard, such as giving a patient a double dose of medication and asked the patient to verify whether the mistake had been made or not. Although the patients did not notice the error themselves, they could participate by confirming the nurses’ suspicions.

## 4 DISCUSSION

The aim of this study was to explore nurses’ perceptions and experiences with regard to patient involvement of potential relevance for patient safety. The study contributes to the research field by addressing the nurses’ perspective in contrast to much previous work that has concerned patient views. Further, the study provides insights into how patient involvement for safer care can be achieved in the provider‐patient interaction. In general, the nurses expressed positive attitudes to patient involvement and believed it could have a positive impact on patient safety. However, patient involvement does not occur by itself. Rather, both patients and healthcare professionals must take responsibility if patient involvement for safer care is going to be realized.

The nurses in our study emphasized the importance of their own initiatives to achieve patient involvement. They stated that healthcare professionals can facilitate this involvement by initiating dialogue and inviting the patients to ask questions. Our findings are consistent with previous research from the patient perspective, which has shown the importance of healthcare professionals encouraging patients to speak their opinion (Davis, Koutantji, & Vincent, 2008; Entwistle et al., 2010; Rainey, Ehrich, Mackintosh, & Sandall, 2015). It has been suggested that patients, due to imbalance of power and health literacy, are unwilling to speak their mind if they fear negative or judgemental reactions from the providers, or being ignored or not taken seriously (Davis, Sevdalis, Jacklin, & Vincent, 2012). This is supported by our findings from the nurses’ viewpoint, because the nurses highlighted the relevance of building a trustful relationship with the patient by actively listen to them and encourage them to express opinions and ask questions.

Further, the nurses pointed to the importance of providing individualized information to the patients. In a previous Swedish study examining facilitators and barriers to patient safety, nurses expressed that providing well‐structured information to patients is a facilitator for patient safety (Ridelberg et al., 2014). Further, research from the patient perspective has highlighted the value of patients understanding of information for them to participate in their care and to make informed decisions (Davis et al., 2012; Eldh, Ehnfors, & Ekman, 2006; Longtin et al., 2010). Patients who have been comprehensively informed are also more likely to feel confident and trust their own decisions (Forsyth, Maddock, Iedema, & Lessere, 2010; Longtin et al., 2010). Provision of appropriate and sufficient information in a supportive environment are key points in patient involvement (Larsson, Sahlsten, Sjostrom, Lindencrona, & Plos, 2007). Patients who have access to information on their health and care are more willing and able to be involved in safety issues (Forsyth et al., 2010; Iedema et al., 2012). It is likely that patients who receive adequate information become more knowledgeable about what to expect from nursing activities, treatment and care, which enables them to detect potential deviations of relevance for patient safety.

The hindering factors associated with patient involvement for safer care that we found in this study are largely consistent with the barriers identified in research on patient involvement from the patient perspective (Howe, 2006; Iedema et al., 2012; Larsson, Sahlsten, Segesten, & Plos, 2011). With regard to shared decision making in health care, Joseph‐Williams, Elwyn, and Edwards (2014) concluded in a systematic review that patients’ participation depends on their knowledge (about the condition, options for care, outcomes and personal preferences) and power, that is, perceived influence on decision making. The two factors, knowledge and power, are in turn influenced by interpersonal patient‐provider factors, patient characteristics, trust and time allocated for discussions. Assessing nurses’ opinions of factors influencing patient safety in general, Ridelberg et al. (2014) found factors relating to both patient interactions and healthcare providers skills and feelings to be potential barriers for patient safety.

It has been suggested that nurses believe patients lack sufficient medical knowledge, making it necessary for nurses to retain power and control (Henderson, 2003). Grimen (2009) has highlighted the interconnection between power and trust, arguing that many healthcare professionals fail to recognize the power associated with professional autonomy, which makes equal dialogue between patients and healthcare professionals unrealistic; patients are in an inferior position vis à vis healthcare professionals. Hence, being a patient is to trust that professionals know what they are doing and to temporary delegate power to them. On the other hand, knowledge and power is a two‐edged sword, which if used wisely in a patient‐provider encounter, can foster mutual respect for the knowledge possessed by both patients and healthcare professionals (Eldh, Ekman, & Ehnfors, 2010).

Ignorance of this provider‐patient power imbalance could make nurses resistant to patient involvement because they do not believe in and inform themselves about the patients’ opportunities to make informed contributions. This in turn contributes to creating a culture of professional defensiveness towards patient involvement (Henderson, 2003; Howe, 2006). Some nurses in this study mentioned that active patients can be time consuming and that too much time is wasted on explaining irrelevant matters to the patients. Communication with patients cannot always be prioritized, because nurses also need to focus on taking care of risk situations and complete tasks (Tobiano, Marshall, Bucknall, & Chaboyer, 2016). As pointed out by Ekdahl, Hellström, Andersson, and Friedrichsen (2012), the remuneration system used in Swedish health care favours treating a large number of patients, which leads to time restrictions and insufficient time for many patients. Time barriers exist not only in Sweden. In a study on patient involvement conducted in 15 European countries, time spent with patients and communications were perceived as the most important areas for improvement of patient involvement (European Commission, 2012). Organizational factors such as time constraints (Bolster & Manias, 2010; Entwistle et al., 2010) and lack of continuity in care (Unruh & Pratt, 2007) have previously been suggested to have a negative impact on patients’ active involvement in safety work. For individual healthcare professionals to be able to invite patients to be involved in their care, as suggested by the nurses in our study, requires a shift in the healthcare system to allow more time for conversations with each patient. Our study also pointed to the relevance of the nurses’ ambivalent feelings towards patient involvement. Perceiving that one's professionalism is questioned could hinder providers from actively involving patients in some situations.

### 4.1 Limitations

This study has several shortcomings that must be considered when interpreting the results. The recruitment strategy could have led to a bias towards participation by nurses who were more interested in patient involvement and/or patient safety issues. The importance of patient involvement has recently been highlighted in Sweden. This might have led to the participants providing more positive answers in the interviews because they want to provide responses that are somehow politically correct. On the other hand, the interview guide was constructed to give the responders the opportunity to answer in general terms rather than revealing their personal opinions.

Nineteen individual interviews with registered nurses and nurse assistants working in different types of healthcare settings were conducted. Various ages, work experience and types of patients contribute to a large variation in the sample. This heterogeneity increases the possibility of viewing patient involvement for improved patient safety from different angles, which can be considered a strength in the study. Inclusion of male nurses in the sample would have increased the heterogeneity further. However, the lack of male participants was deemed acceptable since 88% of registered nurses and 84% of nurse assistants working in Swedish health care are female (SALAR, 2015). Transparency was sought by describing the sampling procedure and data analyses in detail.

During the interviews and data analysis, it became evident that the nurses did not always share our definition of patient safety. Although the official definition of patient safety was read to the participants at the beginning of the interview, they tended to interpret the concept more broadly to encompass various aspects of health care in general. This was especially common among the nurse assistants; they provided examples that had more to do with regular health care provision than with patient safety as defined. We found this interesting and did not want to interrupt to impede the nurses’ willingness to tell stories they found important. However, our findings primarily relate to various aspects of patient safety, as defined in this study.

## 5 CONCLUSIONS

We found that nurses are in general positive to patient involvement and believe it can contribute to increased patient safety. The nurses believe that they can influence patient involvement and that they have a responsibility to do so, but that the patients are responsible for being active in meetings with healthcare professionals. Patient involvement also depends on a well‐functioning provider‐patient interaction. The finding also suggest that healthcare professionals need support from the healthcare system to achieve patient involvement of relevance for patient safety.

## CONFLICT OF INTEREST

The authors declare that they have no conflicts of interests.
